# A safe and effective surgical approach for congenital tracheo-oesophageal fistula in adults

**DOI:** 10.1093/icvts/ivaf097

**Published:** 2025-04-16

**Authors:** Jiangshan Ai, Lianzheng Zhao, Huijiang Gao, Yucheng Wei

**Affiliations:** Department of Thoracic Surgery, The Affiliated Hospital of Qingdao University, Qingdao, China; Department of Thoracic Surgery, The Affiliated Hospital of Qingdao University, Qingdao, China; Department of Thoracic Surgery, The Affiliated Hospital of Qingdao University, Qingdao, China; Department of Thoracic Surgery, The Affiliated Hospital of Qingdao University, Qingdao, China

**Keywords:** Oesophagus, Congenital, Tracheo-oesophagea, Surgical approach

## Abstract

Congenital tracheo-oesophageal fistula in adults is a very rare condition; there is no standard surgical procedure for adult patients. This case report describes a successful surgical treatment of an adult congenital tracheo-oesophageal fistula using a central sternotomy approach and summarizes the specific surgical steps. We believe that this approach is a safe and effective method for treating such disease.

## INTRODUCTION

Congenital tracheo-oesophageal fistula in adults is a very rare condition. Patients frequently present with postprandial coughing symptoms and recurrent pulmonary infections since childhood [1]. Due to delayed diagnosis, the condition may progress to its adult stage, even culminating in irreversible pulmonary damage [2]. Surgical treatment is the only method of cure; however, there has been no research to develop a standard surgical protocol yet.

## CASE REPORT

**Figure 1:**
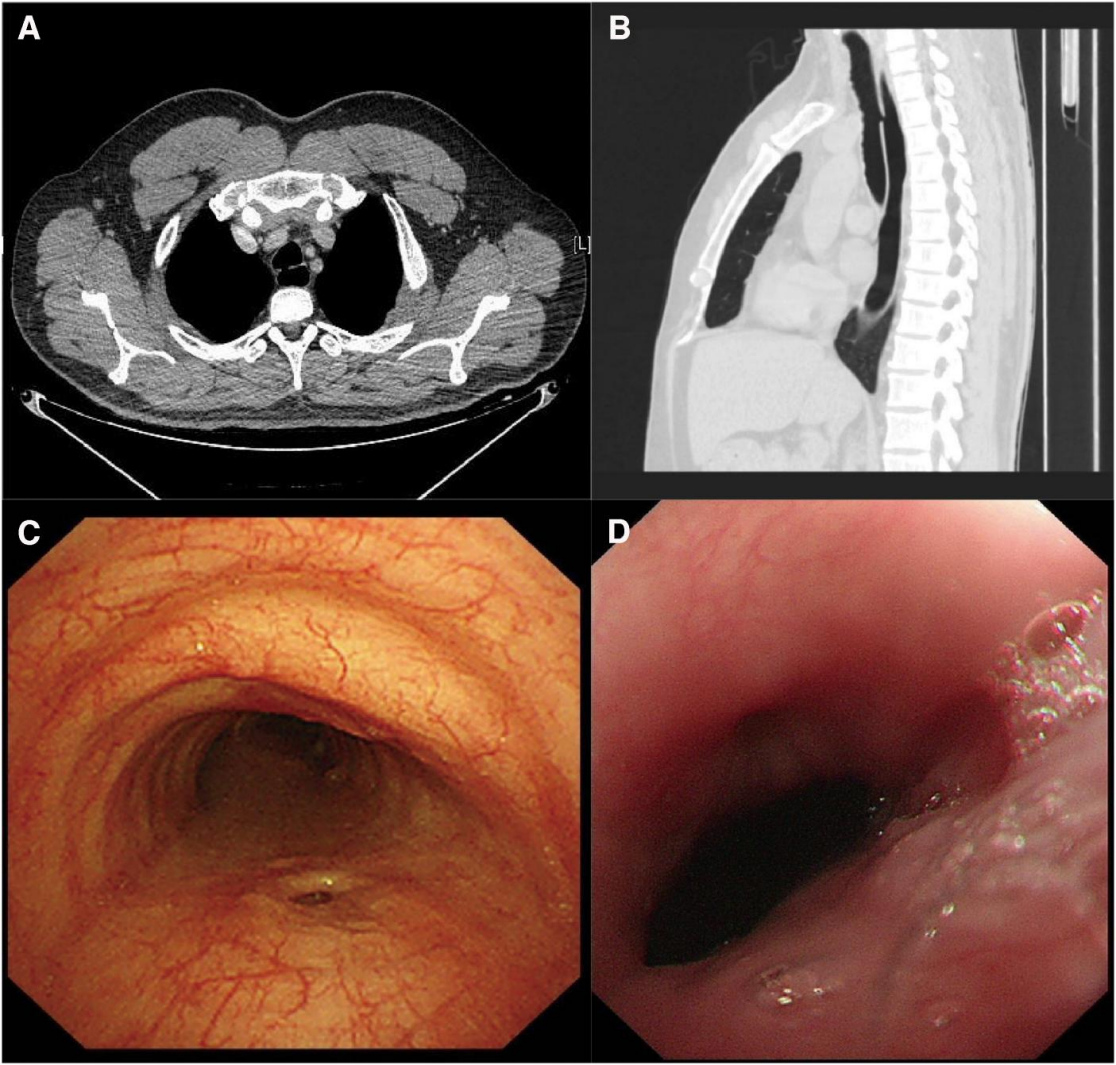
(**A**) Axial chest CT scan of the patient shows a communication between the trachea and oesophagus. (**B**) Coronal chest CT scan of the patient shows a communication between the trachea and oesophagus. (**C**) Endoscopic examination of the patient shows a fistula at the membranous portion. (**D**) Oesophagoscopic examination of the patient shows an oesophageal fistula.

A 35-year-old male, non-smoker, presented to our hospital with a cough after eating. The patient has had recurrent pneumonia since childhood without a clear cause. Chest computed tomography (CT) scan showed a communication between the trachea and oesophagus (Fig. 1A and B). A bronchoscopy showed an elliptical fistula approximately 5–8 mm in diameter at the upper trachea, 7 cm from the vocal cords, which changed in size with respiration (Fig. 1C). An oesophagoscope showed a fistula ∼1 cm in size 20 cm from the incisors (Fig. 1D). The patient was diagnosed with congenital H-type tracheal-oesophageal fistula.

**Figure 2:**
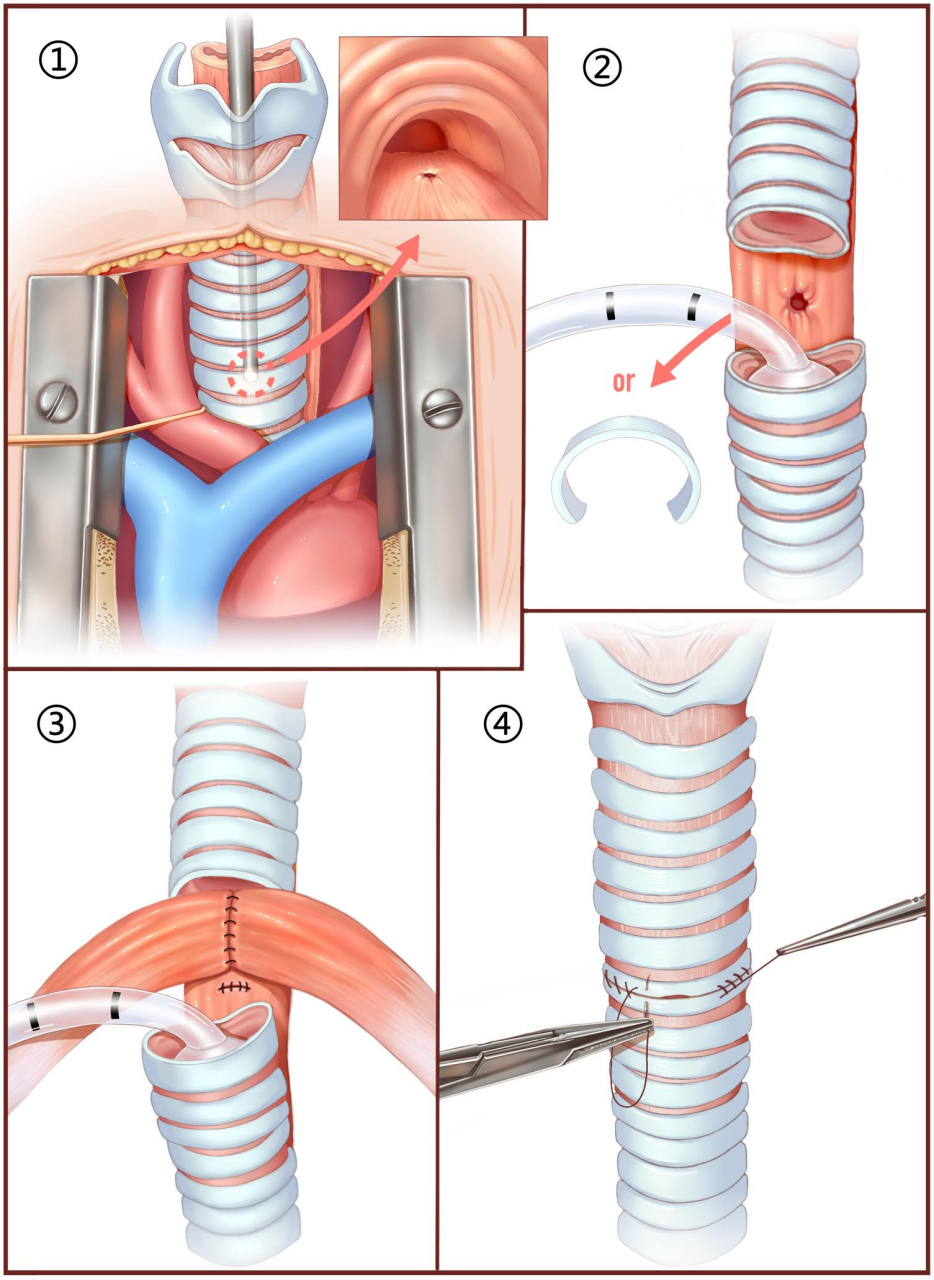
Illustrated surgical procedure.

After completing a thorough preoperative examination to rule out contraindications, the patient was given surgical treatment and the surgical procedure was divided into 4 steps (Fig. 2): (i) a central sternotomy was performed to expose the trachea, and bronchoscope was used to assist in locating the fistula; (ii) a tracheotomy was performed at the level of the fistula and dissected it accordingly, based on fistula size and the degree of local infection, the corresponding tracheal ring may also be removed. The tracheal stump is intermittently intubated for assisted breathing; (iii) the oesophageal fistula was repaired by layer-by-layer suture, while the 2 lateral subhyoid muscles are detached and fused into a muscle flap to cover the fistula; and (iv) the trachea was anastomosed and a sealing test was performed (Video 1).

The patient had a smooth recovery after surgery, an upper gastrointestinal contrast examination was performed 13th day after the operation. The contrast agent passed through the oesophagus smoothly, and no external leakage was seen. The patient has been followed up for 1 year, with a good quality of life and complete disappearance of postprandial coughing.

## DISCUSSION

Congenital tracheo-oesophageal fistula in adults is indeed a rare condition, and patients often undergo multiple misdiagnoses. When a patient experiences persistent coughing after eating and recurrent pneumonia, it is important to consider the possibility of this disease. The CT sagittal reconstruction image should be promptly conducted to identify the fistula, and confirmation of the diagnosis can be achieved through oesophagoscopy and bronchoscopy.

According to previous study, there is a risk of recurrence after surgery for congenital tracheo-oesophageal fistula [3], so it is necessary to fully repair the fistula both in the trachea side and oesophagus side. In this particular case, given that the fistula is situated at the level of the manubrium sterni, we opted for a thoracic approach. However, if the fistula located slightly more proximal, a cervical approach would undoubtedly present a less invasive and equally viable alternative [2]. There have also been reports of cases where the fistula was closed using stapler during a thoracoscopic procedure [1], which is suitable for fistulas located entirely within the thoracic cavity, and the fistula should be a long H-shape communication; otherwise, it may result in excessive tension at the anastomosis site and increase the risk of postoperative anastomosis fistula.

In this case, the fistula was located posterior to the sternum, therefore, we used the central sternotomy approach, although there is an unavoidable surgical trauma with sternotomy, this technique can provide a good field of view and exposure to complete the repair of both side of fistula and avoid excessive surgical risk. Future improvements in surgical techniques could involve making a ministernotomy to reduce the incision size and minimize trauma. It is worth noting that the precise positioning of the fistula and the tracheal plane is determined by the intraoperative bronchoscopy.

In this study, the postoperative recovery of the patients was smooth, with no complications. This surgical procedure was safe and effective in this case, suggesting its potential as a viable treatment option for similar patients.

## ETHICAL STATEMENT

The ethical institutional review board of our hospital approved the case report and publication of data (No. QYFY WZLL 28532). The patient agreed that their medical records and treatment information could be used for scientific research and publication of papers, and signed the informed consent form.

## Data Availability

The data underlying this article are available in the article.
